# Cross-Species Affective Neuroscience Decoding of the Primal Affective Experiences of Humans and Related Animals

**DOI:** 10.1371/journal.pone.0021236

**Published:** 2011-09-07

**Authors:** Jaak Panksepp

**Affiliations:** Department of Veterinary & Comparative Anatomy, Pharmacology and Physiology College of Veterinary Medicine, Washington State University, Pullman, Washington, United States of America; French National Centre for Scientific Research, France

## Abstract

**Background:**

The issue of whether other animals have internally felt experiences has vexed animal behavioral science since its inception. Although most investigators remain agnostic on such contentious issues, there is now abundant experimental evidence indicating that all mammals have negatively and positively-valenced emotional networks concentrated in homologous brain regions that mediate affective experiences when animals are emotionally aroused. That is what the neuroscientific evidence indicates.

**Principal Findings:**

The relevant lines of evidence are as follows: 1) It is easy to elicit powerful unconditioned emotional responses using localized electrical stimulation of the brain (ESB); these effects are concentrated in ancient subcortical brain regions. Seven types of emotional arousals have been described; using a special capitalized nomenclature for such primary process emotional systems, they are SEEKING, RAGE, FEAR, LUST, CARE, PANIC/GRIEF and PLAY. 2) These brain circuits are situated in homologous subcortical brain regions in all vertebrates tested. Thus, if one activates FEAR arousal circuits in rats, cats or primates, all exhibit similar fear responses. 3) All primary-process emotional-instinctual urges, even ones as complex as social PLAY, remain intact after radical neo-decortication early in life; thus, the neocortex is not essential for the generation of primary-process emotionality. 4) Using diverse measures, one can demonstrate that animals like and dislike ESB of brain regions that evoke unconditioned instinctual emotional behaviors: Such ESBs can serve as ‘rewards’ and ‘punishments’ in diverse approach and escape/avoidance learning tasks. 5) Comparable ESB of human brains yield comparable affective experiences. Thus, robust evidence indicates that raw *primary-process* (i.e., instinctual, unconditioned) emotional behaviors and feelings emanate from homologous brain functions in all mammals (see Appendix S1), which are regulated by higher brain regions. Such findings suggest nested-hierarchies of BrainMind affective processing, with primal emotional functions being foundational for secondary-process learning and memory mechanisms, which interface with tertiary-process cognitive-thoughtful functions of the BrainMind.

## Introduction

The most intense affective experiences humans ever have are during emotional episodes. All other mammals exhibit similar types of emotional arousals. But do they experience affective states when their external behaviors are intensely emotional? Most interested scholars and the public at large answer, “Obviously they do.” This everyday conclusion is now supported by both behavioral [1] and neuroscientific evidence [2], [3]. However, most careful scholars who scientifically study emotions tend to assume an agnostic stance. Let me only consider a most recent example: Mendl, Burman, and Paul [4], at the beginning of a fine recent paper on the emotional choices made by animals, carefully indicated that the emotional behaviors of animals “may or may not be experienced consciously.” An accompanying commentary on that article highlighted epistemological ways out of such conundrums, by basing arguments on triangulated evidence from affective neuroscience [5]—relating i) brain mechanisms, to both ii) behavior and iii) experiential-affective analyses (see below). Behavior-only research cannot achieve definitive conclusions, since it has no direct access to underlying affective infrastructure of certain brain mechanisms. Thus, if we just analyze behavior, we have no empirical way out of the conundrum of belief-based conclusions. With the inclusion of neuroscience, especially direct evaluation of the affective properties of the underlying brain systems, we can base our conclusions on evidence, and the position advanced here is that abundant data has long indicated that animals do experience their emotional arousals. In short, activation of various brain systems can serve as “rewards” and “punishments” in various learning tasks [2]. Thus, we know approximately where affective states are generated in the brain although we do not know exactly how. Such subcortical loci of control allow us to entertain the idea that a study of emotional circuits in animal brains can illuminate the primal sources of human emotional feelings. But the relevant brain and behavioral/psychological sciences have yet to embrace such conclusions, and agnosticism prevails. Thus, this paper is premised on the fact that it is within the brain mechanisms of unconditioned emotional behaviors where we find the strongest empirical evidence for the emotional feelings of animals.

**Figure 1:**
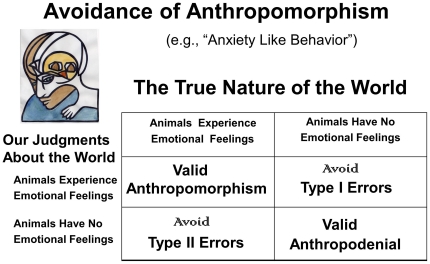
A truth diagram of anthropomorphism. A truth diagram relating how we need to think about the possible affective nature of animals (The true nature of the world) and our corresponding scientific judgments about the world. Most of the 20th century was spent believing that the right lower corner was the correct place to be philosophically so one could avoid Type I errors, namely concluding something that is not true to be scientifically correct. This led to discussions of “anxiety-like” behaviors in animals as opposed to fear in animals. This article is premised on the data-based conclusion that individuals who are conversant with the relevant data are wise to situate themselves in the upper left quadrant, since that way we can avoid Type II errors, namely the failure to detect a real phenomenon, because we have false beliefs, or inadequate methods to evaluate the presence of a phenomenon.

Empirical resolution of the perennial dilemma of subjectively experienced emotions in other creatures (a form of phenomenal consciousness) raises important issues for animal welfare debates and provides scientific paths for working out the neural mechanisms that generate valuative internal experiences in other animals. That knowledge could guide understanding of the foundations of our own brains and minds. Of course, there continues to exist a widespread fear of anthropomorphism in the cross-species brain sciences (Figure 1), which may no longer be as wise as it seemed just a few decades ago [2]. This paper discusses the kinds of evidence that currently provide the most robust scientific support for the existence of subjective affective experiences in the animals we study. Namely, if artificial experimental arousal of brain networks that control emotional behaviors can also routinely serve as ‘rewards’ and ‘punishments’ that can guide learning, then the evidence for certain types of positive and negative experiences in their *brains*, may we say *minds*, is close to definitive. That is, unless one could routinely demonstrate that ‘rewards’ and ‘punishments’ in humans are typically unconscious—a data base that does not exist. Thus, the goal of this essay is to discuss whether other animals are feeling creatures not just on the basis of reasoned arguments (which is common, see the Denouement at the very end of this paper), but also in the context of the most relevant neuroscientific evidence. Thus, the following conclusion is empirically justified: At the very least, all other mammals experience their emotional arousals.

## Analysis

### The Affective Sources of the BrainMind: Cross-Species Neuroevolutionary Perspectives

The behavioral data for animal emotions have been definitive for a long time, from Darwin (1872) to Mendl et al this past year [4], so to speak. However an equally important but comparatively neglected issue is whether animals have the kinds of brains that can engender subjectively experienced states. Such “mind stuff” can only be scientifically penetrated with functional neuroscience. To reiterate the most critical point already noted: If one can demonstrate that brain networks that participate in generating coherent emotional reactions also mediate ‘rewarding’ and ‘punishing’ states *within* the brain, without employing any external objects such as food and water to train animals, we would have robust evidence for locating central processing stations for certain types of affective experiences in specific brain regions and circuits. Further, if certain underlying circuit attributes in animals (e.g., neurochemistries) modulate the within-brain processes that lead various external events to be rewarding and punishing in both animals and humans, we will have fulfilled another critical experiential prediction. There are abundant investigations of drug addictions (especially for morphine and various psychostimulants), that will not be summarized here, that satisfy that criterion. Further because of evolutionary homologies in the underlying subcortical brain mechanisms in all mammals, the above knowledge offers direct predictions to qualitative human experiences following similar brain manipulations. In other words, if our predictions about changing internal feelings in humans, derived from the animal data, are supported by human self-reports, as has often been the case [2], [6], we have additional reasons for confidence that both humans and animals are having similar (albeit not identical) experiences.

Indeed, the above criteria, based on many studies of electrical stimulation of the brain (ESB) and chemical stimulation of the brain (CSB), have supported the existence of emotional feelings in animals for many years; such stimulation can trigger emotional-behavioral episodes, yielding brain states of various kinds that also serve to motivate various learned approach-and-avoidance behaviors, providing abundant evidence for positive and negative feelings in animals. This gets us as close as we can presently get *scientifically* to the mechanisms that generate affective feelings in mammalian brains. In addition, if humans report distinct emotional experiences from such brain sites, we have additional *prima facie* evidence for corresponding types of emotional feelings in animals. It could be supposed that the actual experience of affective states is achieved by higher brain mechanisms that are activated by emotional arousals, but that would have to be deemed a “second best” hypothesis for it becomes un-parsimonious by adding an additional loop of complexity to the overall equation.

Why has substantive knowledge about animal emotional feelings had so little effect on the debate about the existence of subjective experiences in animals? Especially when such knowledge may clarify the sources of affective emotional experiences in humans? This appears to be due to a sustained bias during most of the 20^th^ century that the internal experiences of animals are outside the realm of rigorous scientific inquiry [7]. Of course, the attitude of skepticism is deeply valued by many scientists, including myself. However, there are many historical antecedents where, because of this precious attitude, critical lines of existing evidence were devalued without counter-evidence and hence new evidence-based conclusions were not adequately considered, and hence have been long neglected. This has often slowed down the progress of science because of prevailing biases against transformational concepts that are unwelcome in the Zeitgeist. For instance, one common bias among behaviorists of the 20^th^ century was that the brain did not need to be understood to have a coherent science of behavior. That attitude may have seemed fair enough before modern neuroscience, but because the study of the “black-box” was long marginalized, when neuroscientific knowledge suggested that an understanding of emotional states was ripe for the picking, there were few to harvest the low hanging fruit.

Now that there is abundant relevant neuroscience in the field (aka, behavioral neuroscience), which has quite consistently provided evidence for the rewarding and punishing nature of brain circuits that mediate emotional behaviors [2], [3], [6], affective constructs are still not widely used because of the continuing fear of anthropomorphism, making it a still prevailing attitude that presently is evolutionarily unfounded (see Figure 1). The failure of affective concepts to become common currency in animal research has, I would argue, had negative influence on cross-disciplinary integrations, which could have rapidly advanced fields like biological psychiatry, through the recognition that emotional feelings were ancient functions of medially situated brainstem regions. Instead, when cognitive neuroscientists became intensely interested in emotions with the ready availability of modern brain imaging in the mid 1990s, most investigators accepted the traditional view that not only was the neocortex the seat of conscious thought, but also of emotional feelings. As a result, emotional feelings were not granted to animals, for they were commonly deemed to be a form of thought, and affective and cognitive processes were envisioned to be completely interpenetrant in higher brain regions that generated certain higher cognitive processes such as frontal cortical regions.

**Figure 2:**
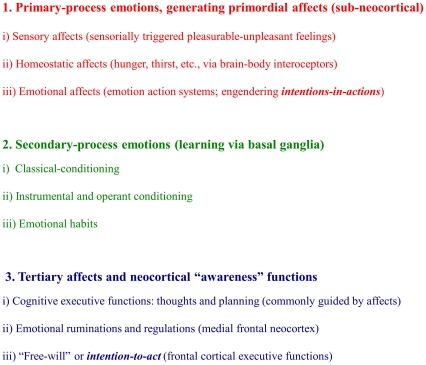
Levels of control in brain emotion-affective processing. A summary of the global levels of control within the brain 1) with 3 general types of affects (red), 2) three types of basic learning mechanisms (green), and 3) three representative awareness functions (blue) of the neocortex (which relies completely on multiple levels of integration, with descending controls down through the basal ganglia to the thalamus, looping back to neocortex) before it can fully elaborate both thoughts and behavior).

Indeed considering the evolutionarily layered nature of brain organization, I will argue that one can readily use cross-species anthropomorphic reasoning at *primary-process* subcortical MindBrain levels, albeit not at the *tertiary-process* neocortical levels, as summarized in Figure 2. These primal evolutionary concepts will be discussed more extensively after a thumbnail sketch of the recent history of the field that has generally slowed the acceptance of animal emotional feelings, as a gateway to understanding both human and animal emotions, as a key topic of experimental inquiry.

### Historical Perspectives

As already noted, Charles Darwin (1809–1882), who wrote what is widely deemed to be the first modern scientific treatment of the subject of emotions, set the stage by intuiting that animals have emotional lives not all that different from our own. With his principle of evolutionary continuity of mind among animals, he suggested that understanding animal emotions could scientifically illuminate our own emotional lives [8]. Darwin had no problem in imputing emotional feelings to other animals.

Darwin's view, however, did not percolate clearly up to the present-day, namely to the neurobehavioral and neuropsychological sciences. Indeed, the theories of many subsequent thinkers, starting prominently with William James, focused on the possibility that higher cognitive regions of the brain mediate our emotional feelings, not just the many thoughts that accompany our emotional arousals. Thus, many scholars at present continue to believe that emotional feelings are a subset of cognitive processes, as did scholars over a century ago. Indeed, the special whole issue of *Cognition & Emotion* devoted to this topic entitled “How distinctive is affective processing” (published in 2007, vol 21(6) edited by Andreas Eder, et al.) generally supports the conclusion that affects are just a subset of cognitive type brain activities, namely based on sensory information processing principles as opposed to intrinsic bodily emotional states (i.e., specific forms of unconditioned responses).

The most famous of these affective-cognitive conflations was advanced in 1885 when William James (1842–1910) [9] and Carl Lange (1834–1900) [10] suggested that emotional feelings merely reflect cortical-cognitive ‘readouts’ of peripheral-unconscious autonomic arousals that occur in our bodies when we exert ourselves in emergency situations—for instance, running away from bears. In this interpretation, bodily information reaches the sensory regions of the cerebral cortex, where the sensations of bodily arousals are transformed into emotional experiences. In effect, emotion-related bodily commotions were integrated into emotional feelings by higher mental processes. Among many scholars, this vision of emotionality served to bring into question the existence of emotional feelings in other animals because they have so much less higher “cognitive brain” matter (i.e., neocortex) compared to humans. But all this happened before we understood the evolutionary construction of the brain, and the recognition that many vast emotional integrative networks, especially for unconditioned emotional responses, were built into the subcortical structure of all mammalian brains during the long course of brain evolution.

This type of neocortical ‘readout’ hypothesis/opinion has survived the test of time but not the test of experimental evidence—in short, it is still widely discussed and believed without hardly any critical (*causal*) evidence to support it, even though brain-imaging *correlates* can be and often are used to support that archaic conclusion. The James-Lange theory became ingrained in psychological science belief systems long before anyone knew much about the emotional networks of mammalian brains, and there it seems to remain, a well-fossilized construct. There were compelling challenges as far back as the 1920s [11], never empirically refuted. Thus, in the emerging brain sciences of the 1970s, the view that we cannot empirically study the emotional feelings of other animals, because they have relatively little neocortex, remained the prevailing view, albeit the topic itself was rarely discussed in neuroscientific circles. Thereby traditional skepticism and agnosticism continued to prevail as the guiding principles in most rare discussions of the topic.

Partly, this stance may have also reflected the widespread rejection of psychoanalytic theory as a scientific way to conceptualize the mind in that era. Although Sigmund Freud (1856–1939) had spent the first decade of his career as a neuroscientist (for a full translation of Freud's neuroscientific contributions, see Solms [12]), his theories, along with those of many of his followers, had made emotions the centerpiece of his psychoanalytic theories and therapies. The failure of such ideas to be subjected to rigorous empirical evaluation, along with the rise of the cognitive and neuroscience revolutions, also diminished the importance of emotions as a topic for experimental study because it was deemed too difficult a problem to solve—namely, how could we ever really know what other animals experienced?

It is noteworthy that Freud repeatedly recognized that a lasting understanding of the mind and emotions, could not be achieved without neuroscience. He often remarked that we could not make sense of affective feelings until we came to terms with the inbuilt “instinctual” nature of emotionality. Freud often claimed that affective states were never unconscious; they were, by definition, always experienced. But he recognized that an empirical-neuroscientific understanding of emotions and other mental-experiential features of the brain could not be achieved in his era, and he decided not to share his speculative neural theories, only later discovered in his posthumously published *Project for a Scientific Psychology*. But soon thereafter, behavioral scientists definitively denied that it was empirically possible to study mental events in animals scientifically, and the book was closed on such topics for a long time. It is only slowly opening, and usually only with regard to their self-evident emotional behaviors, as Darwin recognized, but not their emotional feelings.

As a result, Darwin's famous dictum [13] that the differences in the mental lives of animals are “one of degree and not of kind” never served as a jumping-off point for the scientific understanding of human emotional feelings by studying explicit animal emotional actions, with but a few exceptions (e.g., MacLean [14] and Panksepp [2]). The lack of attention paid to the affective lives of other animals, as opposed to simply their emotional behaviors, by scientists was not simply because Darwin's complete view was rather more subtle than the fragment shared above: “There can be no doubt that the difference between the mind of the lowest man and that of the highest animal is immense….Nevertheless the difference….great as it is, certainly is one of degree and not kind” ([13] p. 127). Now we can be confident that the major degrees of cognitive differences have arisen from higher brain encephalizations, while affective feelings are largely sub-neocortical brain functions.

In sum, the continuing lack of explicit work and discussion in scientific annals about the neural nature of emotional feelings in animals was based on the generally accepted ontological view that the subjective lives of other organisms were impenetrable, while their emotional behaviors were not. Thus, a cross-species evolutionary approach to studying the *bodies* of animals was welcomed, but their *minds* were neglected. If for no other reason than contextualizing the present arguments, it is important to be clear about the forces that led science to neglect the emotional feelings of animals.

So let me flesh out the above history in modest detail. Despite promising initiatives early in the 20^th^ century, such as the work of Walter Cannon [11] in physiology and McDougall in psychology [15], discussions of the mental aspects of brain functions that control animal behaviors withered. With the move toward ultra “positivism” in philosophy (e.g., the so called Vienna School) which reinforced the behaviorist revolution, mental concepts in scientific discussions of animal behaviors seemed less important than ever. Behavior could be operationalized, but mind could not. The easiest behaviors to study systematically in the laboratory were those shaped through ‘reinforcement’ contingencies in various automated learning paradigms—classical conditioning, and training of conditioned lever presses and such. This led to a radical behaviorism, and B.F. Skinner (1904–1990) put it bluntly: “The ‘emotions’ are excellent examples of the fictional causes to which we commonly attribute behavior” [16]. It is no secret that to this day many, perhaps most, behavioral neuroscientists deny that we have scientific access to the emotional mind of animals, albeit there are many strands of thinking outside the scientific mainstream that appreciate the likelihood that animal minds are real and can be understood (see the final “Denouement” section of this paper).

Nobel Prize-winning ethologist Niko Tinbergen (1907–1988) put it succinctly and poignantly in his celebrated *Study of Instinct* (1951) [17]: “Because subjective phenomena cannot be observed objectively in animals, it is idle to claim or deny their existence” ([17] p. 5). In the same period, Nobel laureate Walter Hess discovered that rage could be readily evoked by ESB of the hypothalamus in cats. Later in life he indicated that he chose to describe the angry-type attack behaviors as being ‘sham-rage’ because he did not want to have his work marginalized by the behaviorist school. In fact, his unshared personal conviction had been that those rage-like behaviors reflected true experiences of anger. With the transformation of substantial segments of methodological behaviorism to “behavioral and cognitive neuroscience” strategies (starting explicitly in the early 1970s), Hess's original views were accepted as state of the art conclusions (despite demonstrations of the punishing properties of the underlying circuits [2]). And it is clear to all in the field that discussions of animal experiences in academic neuroscience and psychology have remained muted to the present day.

A few ethologists, most prominently Don Griffin (1915–2003) [18], [19], did argue forcefully for *cognitive* mentality (e.g., thoughts) in animals, and a few others have entertained the existence of experienced *emotions* in animals (e.g., see the Don Griffin memorial issue of *Consciousness & Cognition*, March 2005). However, the upshot of the above history is that, at present, most scientists seem disinterested or choose to remain agnostic on such issues. This essay seeks to highlight how abundant cross-species affective neuroscience research, in fact, now strongly supports the everyday insight—“of course, other animals have emotional feelings” without anyone needing to claim that they are *identical* to the evolutionarily homologous human feelings. Evolution is diversity, with homologies highlighting relatedness without any claims about identity.

Thus, the present essay seeks to bring scientific thinking about these issues into line with the weight of evidence indicating that all mammals share not only very similar instinctual emotional behaviors, but that the activities of the underlying brain networks are closely associated with the feelings of raw emotion. The implications of these discoveries are potentially of profound importance for the evolutionary discussions of human minds, the utility of preclinical translational approaches in biological psychiatry and the foundational nature of ethics, as well as the slowly growing appreciation of the evolutionary continuities in MindBrain functions in all mammals, and probably all other vertebrates.

In this vision, the primary-process affective mind emerged much earlier in evolution than our sophisticated cognitive minds. And I will advance the premise that what came first in evolution, namely that which is primary-process, still serves as a critical foundation for what came later, including some of our higher mental abilities. It is likely that our vast cognitive abilities, and those of other highly cerebrated mammals, were constructed upon an affective-emotional infrastructure that all mammals share homologously. Within such a view, many of the presuppositions of psychology, cognitive science, and neuroscience may be turned on their collective heads. Many of our higher mental abilities are comparatively unconscious, meaning unexperienced, for instance, key aspects of cognitive brain functions such as the basic mechanisms of learning and memory. In contrast, the affective foundations are intensely experienced—since they can serve as ‘rewards’ and ‘punishments’ in learning—albeit those psychological states are, at times, hard to translate into words, symbols which more effectively describe external sensory-perceptual abilities than emotional ones.

### The Evolutionary Layering of the BrainMind

First, an explanation of the use of the term BrainMind and MindBrain in this essay: We all know that dualistic thought has traditionally separated brain and mind, but most neuroscientists who consider such issues now accept that mental processes, namely internal experiences, are thoroughly linked to neural dynamics. Hence it may be wiser to have a monistic term, that does not prioritize either mind or brain, but combines the concepts into a unified term (common variants are brain-mind or mind-brain). Perhaps it makes more ontological sense to simply pull them together into a unified concept, where both variants can be used flexibly depending on the type of argument pursued: With the recognition that the brain has retained anatomical signs of evolutionary layerings, perhaps *BrainMind* is better for discussing bottom up issues, while *MindBrain* could be reserved for top-down ones. Since the highest levels of mind (thoughts and plans) are clearly dependent on neocortical functions, they are truly much harder to study *experimentally* and *experientially* in animals than the basic emotional affects. Implicitly experienced cognitive processes have no clear behavioral markers as do measures of affective valence (i.e., rewarding and punishing BrainMind functions that correspond to certain unconditioned response systems of the brain).

It has been challenging to generate a coherent nomenclature for primary-process categories of mind, such as the basic emotions in animals. I have sought to do this most pointedly for the foundational level—the primary-process level of analysis that is the focus of this essay. The primary-process brain mechanisms for emotions are situated very low and medial in the brain (midbrain, diencephalon and related basal ganglia) which affirms their ancient nature in brain evolution. The higher and more forward expansions of the brain provide neural networks for our higher cognitive abilities. Of course, the layering is relative, with many integrative issues in-between that bind the BrainMind into a coherently operating unit.

Still, if we consider such “layered” evolution of brain organization, as many neuroscientists do (although perhaps not favored by behaviorists or cognitivists), then the localization of a variety of emotional circuits in deep subcortical regions (which unambiguously mediate ‘reward’ and ‘punishment’ functions) strongly supports the conclusion that other animals do experience their own emotional arousals. The alternative— that subcortical rewards and punishments are not experienced at all, or that affective experiences arise only by some type of ‘readout’ by higher brain mechanisms—is not consistent with the evidence. For instance, if that were the case, then it would be easier to evoke rewards and punishments from higher brain regions using brain stimulation, but as neuroscientists who have conducted such work have long known, just the reverse is the case. The lower brain systems sustain reward and punishment functions with the lowest amounts of brain stimulation. Indeed, there is no coherent stream of data that discrete activations of *neocortical* functions in animals arouse *any* robust reward or punishment functions. In contrast, the existence of unambiguous experimentally evoked *subcortical* reward and punishment functions, using localized ESB, is vast and definitive. This provides abundant and consistent support for the idea that raw affective feelings are, in fact, a property of certain ancient subcortical midline brain networks in action. However, it does not tell us *exactly* what the animal is feeling, only that the feelings fall in certain categories such as positive and negative affects of various kinds.

Further, studies of animals and humans that have been decorticated—i.e., had the brain's cortex surgically removed—bear out such conclusions: Primal emotional responses are spared, even strengthened [20]–[22]. This also fits with the common observation that people with dementia typically retain emotional responsivity much more than cognitive abilities. In brief, we have long known that not only can we provoke a variety of instinctual (unconditioned) emotional patterns in animals with localized subcortical ESB, but we also know that such evoked states feel good and bad to animals [3], [6], [23], [24]. It is much harder to be clear about the type of feeling that is generated. But it is from these same brain zones that we can evoke the strongest types of diverse self-reports of distinct affective experiences in humans, and the descriptions of feelings aroused generally match the emotional behavioral patterns that are evoked in animals [25], [26]. Further, since we do know that some of the positive effects are discriminated by animals [27] and many can be differentially influenced by direct manipulation of relevant brain chemistries [2], evidence supports the existence of diverse types of rewarding and punishing BrainMind states, not just homogenous positive and negative affective functions.

But is there proof? Scientists, who most value skepticism (i.e., “show me, please”), realize that experimentation *never* proves anything. It only provides the “weight of evidence” for one view or another. From that perspective, we should all now agree that various emotional affective internal experiences have, in fact, been abundantly and empirically validated in other animals. If not, we would have to provide evidence and realistic hypothesis-based argumentation for how environmental ‘rewards’ and ‘punishments’ promote predictable learned behavioral changes. If they do so without arousing brain affective processes in animals, we have a conundrum on our hands, since they routinely have such effects in humans. Thus, at present, skepticism has gone too far, toward the diametrically opposite realm of belief—that something already well demonstrated does not, in fact, exist. In other words, simply saying that certain ‘objects and events’ of the world ‘reinforce’ behavior will not do. “Reinforcement” is not yet a demonstrated brain function; it is a *procedure* to train animals. That *process* in the brain is just a conjecture. The existence of certain affects is not.

It is more coherent, and I would submit, closer to the truth, to say that the concept of *reinforcement* is the name we give to the way the brain's primary-process affective feeling networks facilitate long-term learned behavioral changes. Indeed, such *unconditioned stimulus* and *response* circuits are critical for most of the types of learning commonly studied by behaviorists, to proceed within the brain.

**Figure 3:**
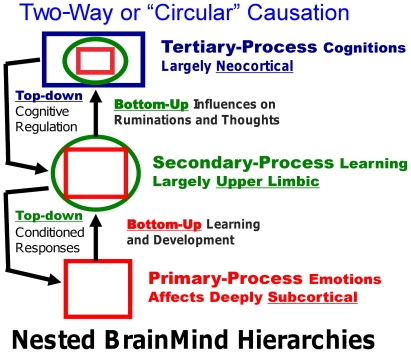
Nested hierarchies of control within the brain. A summary of the hierarchical bottom-up and top-down (circular) causation that is proposed to operate in every primal emotional system of the brain. The schematic summarizes the hypothesis that in order for higher MindBrain functions to mature and function (via bottom-up control), they have to be integrated with the lower BrainMind functions, with primary-processes being depicted as squares (red), secondary-process learning as circles (green), and tertiary processes, by rectangles (blue). The color-coding aims to convey the manner in which nested-hierarchies are integrating lower brain functions into higher brain functions to eventually exert top-down regulatory control (adapted from Northoff, et al. [47]).

This could herald a sea change in the way we envision brain mechanisms of emotional conditioning. Such a view—a modest conceptual readjustment—could put a very different twist on the underlying mechanisms that control commonly studied learning such as ‘fear conditioning’—namely, it may be the raw (unconditional) neural FEAR integration circuits that generate fearful psychological states that attract external information into their orbit. In other words the neuropsychological processes that evolved earlier—e.g., the brain processes that experimental psychologists traditionally call “unconditional stimuli” and “unconditional responses”—are of critical importance for setting up homologous secondary-processes of learning and memory in all species. Such a levels-of-control vision of evolutionary BrainMind layering suggests nested-hierarchy types of emotional organization (Figure 3).

**Figure 4:**
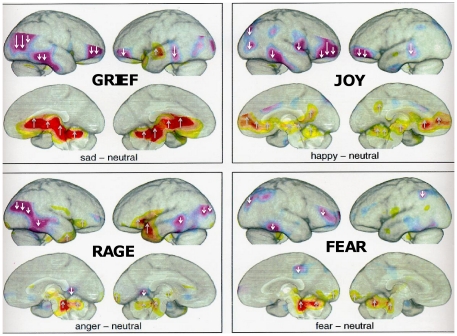
Overview of brain arousals and inhibitions. An overview of brain arousals (reds and yellows) and inhibitions (purples) depicted on lateral surfaces of the right and left hemispheres (top of each panel) and medial surfaces of the corresponding hemispheres (bottom of each panel), while humans experience various basic emotions evoked by autobiographical reminiscing: Upper left: sadness/GRIEF; upper right: happiness/JOY; lower left: anger/RAGE; lower right: anxiety/FEAR (data from Damasio, et al. [38]; overall patterns of activation and inhibition graciously provided by Antonio Damasio). To highlight the directionality of changes, as monitored by changes in blood flow, inhibitions are indicated by downward arrows (predominating in neocortical regions), while arousals are depicted by upward arrows (predominantly in subcortical regions where emotional behaviors can be evoked by brain stimulation in animals).

The primary-process (i.e., basic or primordial) emotions are fine candidates for such functions. However, they are concentrated in such deep and ancient neural networks that there are no generally-accepted experimental strategies to decode their neural nature in humans in any detail. The subcortical organization of emotional affects in our own species is now supported by human brain imaging of basic emotions, as summarized in Figure 4. Animal brain research can achiever higher levels of resolution.

Without a solid cross-species neuroscientific foundation, it may be difficult to make sense of the subsequent mental developments of our species—e.g., the way our cognitive apparatus is often subservient to our emotional feelings. This is inherent in the nested hierarchical view of brain function depicted in Figure 3. Thus, at the foundational level, the differences between human subjective emotional experiences and the mental lives of other mammals may be “one of degree and not of kind” as Darwin surmised, but now we know that the subcortical organization of the emotional systems in mammalian brains is remarkably homologous [2]. An evolutionarily informed cross-species affective neuroscience [3], [23], [24] can now sever the conceptual Gordian knot we have created for ourselves across the years, and solve the mystery of emotional-affective experience in humans as well as other animals. But this Darwinian knife cuts two ways: i) It can return many animals to the ‘circle of affect’ from which they were excluded by scientists, putting additional responsibilities on scientists who wish to conduct ethical research. ii) If animals do experience their primary-process emotions and such primal states of mind serve survival needs, but neuroscientists neglect those aspects of mammalian BrainMind functions, then there can never be any deep neuroscientific understanding of the intrinsic values of human brains. If we continue to neglect the study of emotional experiences in animals, which is currently still common in the field, we may never learn how our human affective feelings are generated, and we will thereby fail to achieve a deep neural understanding of major evolutionary processes that still control our mind and behavior, and our various psychiatric disorders.

### Synopsis of the Classic Evidence and the Needed Integration With Modern Neuroscience

Arguments that we will never be able to scientifically measure the emotional feelings of animals notwithstanding, we have known for a long time that direct stimulation of a variety of subcortical emotional circuits generate ‘reward’ and ‘punishment’ functions in various learning tasks (see research from the era of Delgado, Miller, and colleagues [28], Heath [29] and Olds and Milner [30] in the early 1950s, to the work of MacLean and Panksepp in the 1970s and 80s [14], [23], [31]. There are 21^st^ century indications of a revival of serious research into the affective functions of mammalian brains [6], [32].

If the discovery of central nervous system affective ‘rewards’ and ‘punishments’ cannot be used as a reasonable gold standard for validating the idea that animals do experience their own emotional arousals, I see no credible experimental approach to understanding mental states in other animals nor, as a consequence, ever understanding the neural details of our own raw emotional experiences.

### A Terminological Interlude

The development of a standard scientific nomenclature that can be used to discuss animal minds, in the present case emotional feelings, is bound to be a difficult task. Part of the problem is that all our natural languages are learned skills, tethered to our genetically dictated communicative urges that are molded into an infinite variety of learned nuances by our genetically molded articulatory apparatus. And when it comes to emotional language, there are no rigorous standards that can easily assure agreement. Just think about the different connotations that people have for *sympathy* and *empathy*, which are clearly higher-level emotional concepts. The point is, the science of primary-process animal emotions will surely need a specialized terminology to minimize confusion. And considering the layers of brain evolution (symbolized by the upper-left triune-mind logo of Figure 1), we need distinct labels for primary-process emotions and other affects, which may be the gateway for understanding higher-order affective principles. Before proceeding further, let us contemplate the minimal levels of BrainMind organization that we need to consider (Figure 3).

In neuroscience, primary-process emotional networks must be defined partly in terms of empirically delineated neural and behavioral criteria. For instance, we know that there are subcortical emotional networks that can generate characteristic emotional-instinctual, behaviorally-evident, somatically flexible action patterns accompanied by vast autonomic-visceral changes in the body (i.e., these circuits generate complex *unconditioned responses*) that are initially ‘objectless’—they are activated only by a few unconditioned stimuli. During natural emotional episodes, behavioral and autonomic arousals outlast the precipitating sensory-perceptual inputs, but this aspect has not been well-studied using artificial brain stimulations nor well-controlled studies of natural emotions (i.e., if affective states are sustained by cognitive ruminations in humans, as they surely are, it would be harder to evaluate those levels of control in animals). More speculatively, such emotional arousals that gate/regulate and selectively process sensory/perceptual inputs into the brain, are critical controls in the acquisition of learned behaviors that may help program (and disrupt) many higher brain cognitive/executive functions (cognitions being defined as elaborations by the brain of sensory/perceptual inputs from the external world). With emotional maturation, the developmentally/epigenetically emergent (bottom-up) higher brain functions come to eventually reciprocally regulate (top-down) emotional arousals. Obviously, each level adds complexities to the overall psychobiological equation.

By definition, emotional affects are subjectively experienced, but this tells us nothing about how it all happens in the brain. Although the full emotional package integrates influences from all levels of the BrainMind (Figures 2 and 3), it is clear that the primary-processes—the unconditioned emotional response ssytems—are of critical importance in generating emotional feelings, but it is not clear that anything at this low level of the brain deserves the moniker “cognitive”. To the best of our *experimental* knowledge, primary-process emotional feelings—raw affects—arise directly from genetically encoded emotional action networks (emotional ‘operating’ systems). For instance when such emotion circuits are activated in human brains, as by stimulation of the periaqueductal gray (PAG) of the midbrain, intense feelings are aroused, and they subside rapidly upon termination [26] presumably because cognitive (secondary- and tertiary-process) factors are not sustaining the effects. However, such arousals may progressively lead to endophenotypic shifts in emotional temperaments, as might be evident in psychiatric disorders.

Overall, the data are consistent with a dual-aspect monism view of underlying organization (resembling the dual faces of wave-particle perspectives in physics)—that raw emotional behaviors and their affects arise from the same subcortical neural dynamics. These emotional circuits, generating both emotional behaviors and feelings, anticipate key survival needs, and there is an evolutionary *anticipatory* function for both the behaviors and their primal affective feelings. They tell us promptly whether a course of action may support survival (namely the various positive affects) or hinder survival (the negative-aversive feelings). And in so doing, they mediate what philosophers (e.g., Searle [33]) have called “intentions-in-action” (Figure 2).

But there are other types of affects than the emotional ones that arise from the complex dynamics of brain networks. These others are more closely related to sensory inputs—the pleasures and displeasures of sensation. And besides the *emotional* and *sensory affects*, there are various *homeostatic affects* of the body—the diverse hungers and thirsts of the body that support somatic health. What they have in common is that they all *anticipate* events that will help or harm bodily survival. Pain tells us to back off from certain activities, so as not to injure our bodies any further. These primal affects are ancestral memories of mammalian brains—built into the neural infrastructure to promote survival.

This essay will continue to focus exclusively on those within-brain affects that are here called “primary-process emotions”—namely, those arising from complex action-integrating circuits concentrated in subcortical regions of the brain. In a sense they are most subtle since within-brain intrinsic precipitants may be as common as external triggers, both by local tissue irritations (subcortical epileptic foci) as well as higher cognitive inputs (e.g., ruminations mediated by medial frontal cortical regions [34], [35]). Thus, this essay summarizes affective neuroscience perspectives on *primary-process* emotional affects of mammalian brains that seem to unconditionally arise from the evolutionarily integrated, primordial “instinctual” emotional operating systems of the brain that regulate unconditioned emotional actions, which may be more important in guiding simple emotional learning (e.g., fear-conditioning) than is currently recognized. This essay also looks at *secondary-process* emotions arising from conditioning, both classical and instrumental/operant. However, with our current scientific tools, we can barely touch the *tertiary-process* emotion-cognition integrations in animal-models that reflect our capacity to think and ruminate about our lot in life, which are concentrated in medial-frontal cortical regions. We are obviously the most intellectually sophisticated of mammalian species, and thus such higher neuroaffective issues are best studied in humans, but that is not to say that the neocortical-cognitive apparatus is able to generate any affects merely on its own. Its major role is to regulate emotions—sustaining them with rumination and dampening them with various regulatory strategies that rely on cortical inhibition of subcortical processes, what Aristotle called *phronesis*. Thus, the primordial sources of emotional feelings, important as they are, cannot clarify the whole emotional story.

But how shall we label the emotional primes (i.e., the distinct primary-process unconditional emotional response potentials of the brain)? Holistic MindBrain emotional processes—woven from all evolutionary levels of mentation—have diverse vernacular terms, such as anger, loneliness, anxiety, grief, hope, etc., all of which are tertiary-process concepts. Thus, it would be an error to use such terms to label the primary-process subcortical emotional-affective functions, which in my estimation is the most important level for understanding the evolutionary sources of both animal and human emotion—namely, they are the fundamental level of brain organization upon which the rest of the mental apparatus relies [34]. So what terminologies shall we use to discuss that foundational level so we do not indulge in mereological fallacies—the attribution of the cause of a holistic body-brain-mind arousal to a part of the body rather than to the whole?

**Figure 5:**
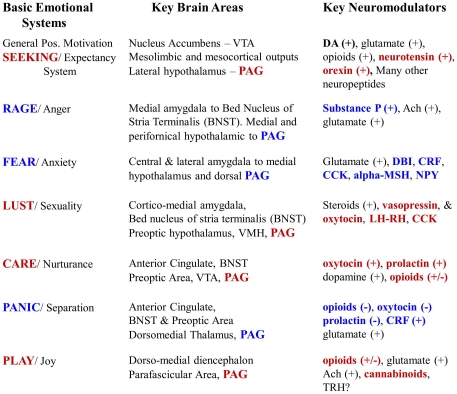
Overview of key neuroanatomies and neurochemistries of the primary-process emotional networks.

This situation mandates a new terminological convention that explicitly acknowledges levels of control but does not lose touch with the foundational importance and nature of raw feelings. Thus, here we follow the terminological choice made a long time ago (full capitalization) for discussing the primary-process emotions of mammalian minds—namely, the SEEKING, RAGE, FEAR, LUST, CARE, PANIC/GRIEF and PLAY systems (for a more complete description of each system, see Appendix S1, with a summarization of key neuroanatomies and neurochemistries in Figure 5). These labels, by using full-capitalization of terms, refer to specific subcortical networks in mammalian brains that promote specific categories of built-in emotional actions and associated feelings. No claim is made of identity with the corresponding vernacular words, although profound homologies are anticipated. Although these systems can never be identical across species (evolutionary diversity rules in all corners of body and mind), the labeling does seek to acknowledge the existence of brain networks that govern various *class-similar* emotional behaviors as well as distinct types of *class-similar* affective experiences in all mammals. Because of evolutionary diversification, we may never be able to objectively describe the precise nature of affective feelings in either humans or animals, but we can at least have confidence in the existence of meaningful similarities in the anatomies, neurochemistries, and psychological functions of these systems across mammalian species. This heuristic will illuminate the mental lives of animals (Figure 4) as well as provide fundamental knowledge for the development of new and more effective medications of psychiatric problems.

In sum, primordial emotional feelings are self-evidently highly interactive with cognitive ‘propositional attitudes’ (each of us feels strongly about *specific* emotion-provoking events we encounter in the world), but such cognitive attitudes are much harder to study rigorously in animals. Clearly, the cognitive mind of animals is less experimentally penetrable at a causal neuroscientific level than the primal affective mind. The above convention for labeling emotional primes may help us sustain clarity of discussion while minimizing mereological fallacies, namely part-whole confusions.

Because the brain is an evolutionarily layered organ, unlike any other in the body, we must also envision how the various ‘hierarchical’ levels seamlessly inter-digitate with each other (Figure 3)—in a sense the evolutionary layers of neural networks are completely inter-penetrant (nested-hierarchies) in the intact brain [2], [34]. Thus, key aspects of lower controls are “re-represented” within the higher levels of control. In this view, during early development the evolutionarily earlier functions (e.g., the unconditioned aspects) provide bottom-up control of higher emotional functions. To put it in other words, lower integrations are functionally embedded in higher functions that emerged later in the evolution of the brain. In this manner, earlier/lower brain functions constrain and guide what the more subtle higher brain functions can achieve, which gradually yield diverse higher-order emotions that are hard to study in animals, from envy to shades of jealousy and resentments. Such thoroughly cognitivized tertiary-processes, thought-related emotions, engendered culturally by social learning are, at present, next to impossible to study rigorously at causal levels and most certainly not in animal models.

From a neuroevolutionary perspective, these ‘beehives’ of *nested hierarchies* allow lower-level controls to maintain some kind of *primacy* in the overall functionality of higher brain networks, albeit perhaps not in the immediate control of behavior when the BrainMind has fully matured. It is likely that the primary processes, upon which organismic-behavioral coherence is based, continue to *anticipate* immediate survival issues, which are then passed on to higher levels via learning-conditioning (*secondary-processes*), thereby providing information for higher working-memory mechanisms, as in dorsolateral frontal cortical regions, that permit tertiary-process deliberative cognitions. Higher forms of consciousness allow humans to plan farther into the future, based on past experiences, than is possible for most other animals. Human planning can use memories that go back toward childhood. This is what is called *autonoetic* consciousness, in the terminology of Endel Tulving [36], namely being aware of one's own life-line from one's personal storehouse of memories of the past and hopes for the future. Some believe that a cross-species affective neuroscience strategy attempts to marginalize those cortically-mediated cognitive-emotional issues. That has never been the case. But if we understand the evolution of the brain, we can more sensitively consider how the higher functional levels are developmentally/epigenetically constructed.

Such hierarchically nested schemes may also help us better appreciate various dilemmas in conceptualizing higher-brain functions and the participation of such functions in psychiatric disorders (see below). Scientific study of animals can only inform us well about the operations of the bottom two levels, with the primary-level being the source of raw (cognitively-unmodulated) affects, and the many unconscious mechanisms of secondary-processing (learning & memory) providing adaptive temporal-spatial patterning of the primary-process affective potentials which arise from lower layers of the BrainMind. What kind of additional affective resolution the tertiary-process level may add is currently unknown, and it is possible that it only mixes the primes ‘neuro-symbolically’ in infinite variations with personal thoughts and impressions to yield the full complexity of our affective lives, constructing profound jealousies, demoralizing shame and guilt, abject desires, and joyous hopes and aspirations—the full human range of affective life from suffering to serene wisdom (*phronesis*, as Aristotle called it; ‘mindfulness’ in modern parlance). This hierarchical vision may also illuminate why investigators working at different hierarchical levels rather commonly do not recognize how their contributions fit into and synergize with different levels of analysis. This evolutionary scheme allows us to envision how the “construction” of higher emotional complexities can even emerge via individual conceptual acts, while not pretending that conceptual acts are the whole story [37]. When we come to the highest layers of the BrainMind, most developed in humans, higher emotion-cognition interactions permit humans the imagination to pursue an almost infinite variety of creative endeavors. However, those higher brain functions may achieve nothing without the ancestral affective foundations of our lower minds—the primary affective processes we share with other mammals.

### The Cross-Species Affective Foundations of Emotional Feelings

Without clear neuroevolutionary approaches, we simply cannot understand the sources of either human or animal emotional feelings and hence how they contribute to emotional disorders and to various issues of animal welfare. In using such cross-species research strategies, we must explicitly recognize that brains, as evolutionarily layered organs, have clear imprints of evolutionary progressions within their anatomical and neurochemical organizations [2], [14], [23]. To summarize, the earliest brain mechanisms remain medially and caudally situated in brains—in their ancestral locations—with most recent developments added rostrally and laterally. Functionally, what emerged earlier remains foundational for later developments, probably “re-represented” in the nested hierarchies noted earlier (Figure 3). The ancient subcortical locus of human feelings has also been found to be subcortically situated (Figure 4), by Damasio and colleagues [38].

As we recognize such nested levels of control within the BrainMind, we should abandon the classic conclusion found in studies on consciousness that subjective experiences arise only from higher MindBrain regions, although our “awareness” of such experiences may be so controlled. Obviously, the lower, phenomenally experienced brain functions (e.g., basic emotions and motivations) are more robustly controlled by inheritance. Higher levels, through social-developmental experiences, add additional layers of control. Lifetime learning can promote increasing ‘plasticity’ of psychological strategies and emotional sentiments that can lead to various moral emotions—from empathy to felt principles of justice. Such accretions of higher mental functions cannot be well-studied neuroscientifically, but the other animals also do seem to have intrinsic moralities [39], as well as capacities to resonate with the distress of others [40]. These moralities are probably expressed in the capacity of animals to develop perceptually driven affective resonance with others—the mammalian social principles that allow LUST to become love, for CARE and PLAY to cement social-support networks and friendships, and PANIC/GRIEF to provide institutional support structures that allow shared grief to help heal the psychological pain that might otherwise cascade into depression.

To summarize the upshot of this vision: In discussing the neural control of emotional behaviors and feelings in humans and other animals, we can usefully parse levels of control into i) primary processes—in behaviorist parlance, the ‘instinctual’ unconditioned stimuli (UCSs) and unconditioned responses (UCRs) of the BrainMind; ii) secondary processes, which reflect the plasticity added by basic mechanisms of conditioned learning and memory; and iii) in some highly cerebrated species, tertiary processes (thoughts, deliberations, etc.), allowing them (and us) to be ‘aware’ of and to reflect upon more primal experiences. A general principle is that mammals are much more similar (albeit never identical) in their subcortical network organizations while being more diversified at higher levels, with the greatest differences occurring at tertiary-process cortical levels.

Clearly, the most recent, tertiary-process layers of MindBrain control can only be well studied in humans. Those higher controls are largely “cognitive” because they rely heavily on the processing of external information. Still, both affective and behavioral neuroscience are more effective in scientifically illuminating the first two levels of control, with studies of secondary controls being especially well defined by studies of the brain mechanisms of fear conditioning (e.g., LeDoux [41]; Maren [42]). In contrast, remarkably few have studied the primary-process feelings and neural organizations [6], [43], and how they may actually promote the learning mechanisms of the brain.

It is important to recognize that the primary-process level is not ‘unconscious’ if one defines consciousness as the ability to have internal *experiences*. From the tertiary-process level the primary-processes may be deemed preconscious, because by itself the foundational level may not be able to be “aware” of its own consciousness—those subcortical emotional networks cannot elaborate what Tulving called *noetic* (knowing) consciousness. The primal level can only mediate *anoetic* consciousness—experience without knowing, but intensely experienced nonetheless. We call this level of experience, affective consciousness [43].

To reiterate, direct ESB-induced activations of these *anoetic* circuits yield diverse ‘rewards’ and ‘punishments’ that guide learning, and in humans, we know that the feelings by such brain stimulations are stronger than those produced by stimulating any other regions of the brain. The secondary learning processes may be largely unconscious, simply the parsing of feelings into diverse temporal and spatial frameworks of individual lives. Tertiary processes are hence mixtures of raw primal experiences and unconscious learning processes, working synergistically in working memory, that yield yet other subtleties (e.g., theories of mind—whereby we are concerned with the thoughts of others). Tertiary processes also allow the higher brain to develop networks of social knowledge, as instantiated in mirror neurons—nerve cells that fire both when an animal/human does something as well as when another animal/human views that something being done.

However, there is currently no data indicating that those higher mental abilities reflect intrinsic brain capacities, as opposed to ones that emerge via social learning.

In any event, to understand how the whole BrainMind operates, we must ultimately consider how higher and lower levels of control participate in the regulation of the whole [34]. We do not yet have good neuroscientific models for that, except for human brain imaging along with some more direct measures of neural activities [44] and, of course, verbal self-reports of experiences. Regardless, all levels need to be existentially integrated for a balanced life. The main tools for achieving full integration of levels scientifically might eventually be through the creative use of massive databases where genetic, neuroanatomical, neurochemical, and functional information can be statistically integrated.

### The Conundrum of Anoetic Affective Consciousness

There are no good reasons to think that emotional-feeling mechanisms have sprung up uniquely in human brains, although some believe it is due to our great capacity for neocortical working memory [45]. The weight of evidence clearly indicates that many affects arise from subcortical brain functions that all mammals share. On that score, cross-species affective neuroscience has already done quite well (e.g., Alcaro and colleagues [35], Damasio and coworkers [38], Mobbs and associates [46], Northoff and colleagues [47], [48] and Zubieta and coworkers [49], to name just a few).

Thus, the real problem is not an epistemological barrier but rather our failure to deal frankly with the emotional lives of the other animals. Stated another way, the problem lies more with the history of our field than with the quantity and quality of the evidence. Indeed, some prominent investigators who traditionally supposed that higher-brain functions generate emotional feelings have now tentatively recognized the critical roles of subcortical loci (e.g., Damasio [50]).

The fact that subjective states cannot be empirically observed as *directly* as behavior should no longer be seen as an insurmountable dilemma. Modern neuroscience can probe such hidden functions of the brain using theoretical strategies that are not all that different conceptually from those that guided the maturation of quantum physics. Certain processes in nature (all the way from the mechanisms of gravity to the feelings of animals) may never be observed *directly*, and they can only be probed and illuminated by focusing on objective external signs, indirect measures, that lead to novel predictions. Measures of emotional vocalizations may be among the best methods to achieve this in predictions that go from animals to humans [51]–[56]. To take one example: Rats make two general broad categories of emotional vocalizations at frequencies that humans can't hear: i) long 22-kHz-type “complaint” vocalizations when confronted by various aversive situations, and ii) short 50 kHz-type “chirps” that signal some kind of positive affect. Clearly those “complaint” networks are situated in affectively negative brain regions such as the dorsal PAG. In contrast, when we evoke the positive “chirps” in rats using ESB, at every brain location where such ‘happy/excited/euphoric’ sounds are evoked, animals will self-stimulate through those electrodes [52]. Thus, we can infer that those emotional sounds directly monitor the affective states of animals.

### Toward a Deeper Psychobiology of the Animal Mind

Epistemological rigor dictates that those theoretical views that can generate the most novel predictions and affirmative observations should rule. That is the time-honored scientific approach to probing the deeper levels of nature that simply cannot be directly observed. For historical reasons, from Cartesian dualism to the dogma of radical behaviorism to the ‘computational theory of mind’ computer-driven cognitive revolution [57], the weight of evidence has not yet had an impact on our discussion of animal feelings, although empirical support for diverse primary-process affective feelings within the brains of all mammals has been available for a long time [2], [3], [6], [58].

That such evidence has been slow to gain acceptance is not, in fact, surprising. Among obvious precedents, consider insights from Galileo to Darwin. A poignant more recent example is the fact that it took the biological community a decade to accept DNA as the hereditary material, despite compelling data provided by Oswald Avery (1877–1955) and colleagues that was published in 1944. The delay arose largely because most scholars believed that only proteins had the requisite complexity to mediate something as complex as genetic inheritance.

Currently, perhaps largely because of the pervasive influence of the James-Lange theory of emotions [59], it is still widely believed that emotional feelings reflect the brain's ability to detect bodily emotional expressions [45], even though evidence at the primary-process level for such an idea remains slim (albeit such processes may be present at learned, secondary-process levels of control [60]). Many investigators still believe that emotional experiences largely reflect higher-brain sensory and homeostatic affective functions–such as those that transpire in frontal, and especially insular, cortices (e.g., Craig [61]). And yet there is precious little causal data to believe that those higher BrainMind levels are the fonts of raw emotional experiences in neural evolution. Indeed, although there is a mass of data implicating the insula in the mediation of pain, the quality of taste, and various somatosensory and interoceptive bodily feelings, this should not be taken to mean that primal *emotional* feelings—RAGE, FEAR, PANIC/GRIEF, PLAY etc. (see Appendix S1) — are constructed there. Although these brain regions routinely “light up” in imaging of the human brain during various emotional tasks, damage to these areas typically does not dramatically impair the capacity for humans to have emotional experiences. As Damasio ([50] p. 77–78) recently noted: “Complete destruction of the insular cortices, from front to back, in both left and right cerebral hemispheres, does not result in a complete abolition of feeling. On the contrary, feelings of pain and pleasure remain. . . Patients report discomfort with temperature extremes; they are displeased by boring tasks and are annoyed when their requests are refused. The social reactivity that depends on the presence of emotional feelings is not compromised. Attachment is maintained even to persons who cannot be recognized as loved ones and friends because . . . of concomitant damage to. . . temporal lobes which severely compromises autobiographical memory.”

And one can also note that electrical stimulation of those insular regions is not especially robust in evoking strong *emotional* states of consciousness in humans, although painful sensory-affective feelings are commonly experienced [62]. In contrast, subcortical stimulations evoke coherent emotional behaviors, including especially strong emotional vocalizations in animals and strong emotional states in humans [25], [26]. Historical reconstruction of the neuronal connectivities of brain areas where stereotactic lesions have been used effectively to treat depressed individuals who have not responded to conventional therapies highlights the convergence of inputs to primal positive emotional networks such as the SEEKING system [63].

## Results and Discussion

### Conclusions

The issue of whether other animals have internally felt experiences that contribute to behavioral control has vexed behavioral science since its inception. Although most investigators remain agnostic on such contentious issues, there is now abundant experimental evidence indicating that all mammals have negatively and positively-valenced emotional networks concentrated in homologous brain regions that may mediate affective experiences when animals are emotionally aroused. The relevant lines of evidence are as follows:

1. Brain scientists can evoke powerful emotional responses by localized ESB applied to distinct brain regions, similar across all mammalian species ever tested. At least 7 types of emotional arousal can be so evoked, and we refer to the underlying systems with a special nomenclature—SEEKING, RAGE, FEAR, LUST, CARE, PANIC/GRIEF and PLAY.
2. These subcortical structures are homologous among all mammals that have been tested. If one arouses the FEAR system, all species studied exhibit similar highly negative emotional responses with differences, of course, in species-typical details.
3. All of these basic emotional urges, from FEAR to social PLAY, remain intact after radical neo-decortication early in life; thus, the neocortex is not essential for the generation of primary-process emotionality.
4. ESB evoked emotional arousals are not psychologically neutral, since all can serve as ‘rewards’ and ‘punishments’ in motivating learning; such affective preferences are especially well indexed by conditioned place preferences and place aversions as well as by animals' eagerness to turn such ESBs on or off.
5. Comparably localized ESB of human brains yield congruent affective experiences—felt emotional arousals that typically appear without reason. In concert with the animal data, this provides robust evidence for emotional experiences in animals exhibiting primary species-typical (instinctual) emotional arousals, and suggests a dual-aspect monism strategy whereby instinctual emotional behavior sequences can serve as proxies for emotional feelings in animals.

Obviously, we can only ask if animals experience something by seeing if such states matter to animals. Will they choose to turn these states on or off? Will they return to or avoid locations where such states were artificially evoked (conditioned place preferences and aversions)? If such intrinsic brain ‘rewards’ and ‘punishments’ are *not* experienced by other mammals, then we truly have a much bigger puzzle, a truly profound scientific dilemma, on our hands: How could rewards and punishments, routinely experienced by humans, control animal behavior through unconscious neural mechanisms? By simply postulating a spooky unconscious process called “reinforcement”? In humans, strong emotions can only be evoked from neural terrain that is demonstrably ancient and homologous in all mammals. Why would such states evoked from subcortical regions of human brains be much different from those in animal brains? Because of neocortical, cognitive ‘readout’ abilities? That is a supposition that creates more conundrums than it currently solves.

Perhaps the biggest contribution of cross-species affective neuroscience research is to decisively return other mammals to their proper status as conscious, feeling beings. This knowledge can provide new information about psychiatric disorders, and a fuller understanding of the neural sources of human affective states (e.g., [64], [65]). But this knowledge also forces us to face ethical dilemmas. The implications of such knowledge for how we live with the other creatures of the world are vast. It is clear that the subcortical powers of our mind—the diverse affective systems that guide our basic living patterns—allow us to feel vibrantly alive as well as gloomy despair. These same systems mediate diverse species typical *experiences* of ‘rewards’ and ‘punishments’, which may be affectively quite similar across species.

One implication of this line of research is that we may never understand the affective depths of our humanity if we ignore our primary-process emotional continuities with non-human animals. This naturalistic, but still novel scientific view of animal minds should help clarify the nature of our own mental lives. If so, it may have enormous implications for the way we raise our children, treat each other and ourselves, and how we shall respect the animals with whom we must find better ways to share the earth.

### Denouement

I write this closing section partly in response to a reviewer of this article who suggested that I had not been fair about the level of scientific work that is being pursued on animal emotions these days. As a point of clarification, I wish to distinguish animal behavior-only and behavioral neuroscience research on emotions, which is a very vast and valuable literature, but not one premised on the direct study of emotional feelings in animals. In contrast, affective neuroscience strategies seek to lay out causal/constitutive strategies to understand the underlying ‘mechanisms’ of affective experiences in mammalian brains. It is noteworthy that primary-process emotions research can be conducted on fully anesthetized animals, for some indices such as appetitive sniffing are still expressed under full anesthesia.

In this essay perhaps I have not conveyed the high level of interest that exists in the study of emotions outside the realm of neuroscience, especially among some animal behaviorists. There are abundant articles on subtle higher-order emotional processes such as empathy, imitation, and fairness, just to name a few, and certainly there is increasing work on animal emotional *behaviors*. Indeed, Marion Dawkins [66] and Franz de Waal [67] have long advocated work on various emotional *behaviors* of animals, while expressing doubt whether we can make a science out of their emotional *states*. If one reads these eminent scholars carefully, it is easy to understand why they hesitate to talk about or even support talk about emotional *experiences*, and implicitly fall back on the agnostic dictum advanced by Nico Tinbergen: “Because subjective phenomena cannot be observed objectively in animals, it is idle to claim or deny their existence” (*vide supra*).

For instance, Dawkins and de Waal have been quite explicit that it is quite impossible to fathom, *scientifically*, the qualitative experiential nature of animal minds. For instance in her wonderful 1993 book *Through Our Eyes Only?*, Dawkins questions whether we can *experimentally* support the contention that animals have true emotional feelings, and does so in all subsequent writings I have read. For instance in her 2001 discussion of “Who Needs Consciousness?” she ends by saying “it is important to be clear where observable facts about behavior and physiology end and assumptions about subjective experiences in other species begin. However plausible the assumption that other species have conscious experiences somewhat like ours is, that assumption cannot be tested in the same way that we can test theories about behavior, hormones or brain activity” ([66] p. S28). de Waal has done the same, with some softening of that perspective (see end of this “Denouement”).

Their nuanced points of view miss my point: A causal neuroscientific analysis has changed the ‘ballgame’. We can now make a variety of testable predictions about the experiential aspects of artificial arousal of brain emotional circuits and how such knowledge can impact human experiences. Now it is no longer a matter of argumentation, but the “weight of evidence”! And that is all science ever has. At present the weight of evidence, based on predictions that have been made, is overwhelmingly for the side of animal affective experiences, with hardly a feather of support for the other side. Scientists, being ultimate skeptics, should honor the rules of the science game, and accept that the neuroscientific evidence now dramatically supports the existence of diverse affective feelings. Acceptance of the evidence opens up the real possibility that we can decode the foundations of human emotions through the study of animal brain functions.

Other scientists working more in the popular vein, especially Marc Bekoff [39], have had no such hesitations; he suggests that our sympathy for nature, along with observations of the nuances of animal behaviors, are sufficient to cross the trans-species mental bridge. I agree as a person, but not as a scientist, especially since the science now provides a solid bridge for individuals who have great emotional sensitivity to other animals to employ scientifically sound arguments rather than their personal convictions. For instance, I remember sitting around a campfire with three friends and visiting members of an elephant conservancy group at the Timbavati Reserve adjoining Kruger Park in South Africa in the fall of 2008. These protectors of the elephants were worrying about how many people keep telling them that other animals, including elephants, do not have emotional feelings, only humans do. I explained how the scientific data from affective neuroscience empirically negates those traditional beliefs, and shared how a strong rebuttal of those ingrained beliefs simply requires the accurate communication of already existing evidence—data of the type already discussed here.

My argument is that sensitive positions that are concurrently liberal at emotional behavioral levels but conservative at phenomenological scientific levels, such as those advanced by Dawkins and de Waal, may still be appropriate for higher-order *cognitive* aspects of animal mental lives (e.g., their possible cognitions and thoughts), but that skepticism should no longer apply to their emotional feelings (*affects*). This is simply because the valenced neural infrastructure of affective states has been well studied with traditional functional neuroscientific methods [2], which provide the scientific evidence for the current arguments. Since this kind of science requires neural investigations, and few animal behaviorists pursue such work, it is understandable that they have not fully weighed the many opportunities to go down to the subjective level *empirically* with the aid of neuroscience. That would not only support their own views about the importance of emotions in animal lives, but also provide an epistemology for further progress. Strangely, they have not yet seized that empirical opportunity, nor recognized the robust experimental strategies neuroscience provides. As a result, the power of a very traditional form of skepticism currently continues to outweigh the evidence even in the minds of the most sensitive investigators of animal behavior.

This does not mean that we can read animal minds in any detail, but we can read the affective arousals and the types of valences that permeate their minds. When integrated with comparable human research—work that is routinely happening in the context of neurosurgery for various disorders (Parkinson's disease, depression, etc.) with therapeutic deep brain stimulation—we can also make concrete predictions, and thereby obtain corroboratory evidence [68] about homologous class-similarities in our affective experiences. The massive subcortical concentration of affective circuits suggests that such BrainMind capacities evolved long before the more recent radiations of mammalian diversity. Species diversity surely means there will be many differences in the types, durations and intensities of emotional feelings among different species and different individuals (including humans), but this does not markedly reduce the possibility of discovering general principles that work across species.

To my knowledge neither Dawkins nor de Waal has considered their “subjectivist dilemma” of other minds, whether in humans or other animals, and recognized how severely it hinders the acceptance of the affective neuroscience perspective advocated here. Thus, the empirical study of emotional feelings has been a workable problem in neuroscience for some time, although few have “taken the plunge” so to speak. There are now abundant cross-species neuro-affective predictions that can be made [2], [65], [68].

On the other hand, various scholars, writing in the popular mode, such as Temple Grandin [1], [63], and most prominently Marc Bekoff [39] accept the reality of animal feelings. But these scholars, and many others with enlightened views, have not pursued neuroscientific research on emotional processes. Hence their important advocacies of seemingly self-evident intuitions are not the same as advancing the rigorous predictions allowed by neuroscientific approaches. Neuroscience, after all, is the only way to verify such constructs and also to illuminate what it means, mechanistically (constitutively), to have subjective experiences. Hopefully my forceful arguments in behalf of affective neuroscience strategies, contextualized in hopefully an accurate portrayal of historical antecedents, will not be envisioned as mere complaints or empirically unjustified anthropomorphism. The intent is to advance the science of mind.

We can finally capitalize on evidence-based neuroevolutionary strategies to understand other minds, not only to illuminate the affective mentalities of other creatures, but also to better understand our own. Why are such endeavors so important? Such knowledge has remarkable potential to advance the understanding of our own emotional feelings, scientifically, perhaps for the first time in human history. With this knowledge we can advance psychiatric insights and aspire to scientifically respect the minds of other creatures—understanding how they could feel their emotions as intensely as we do.

Of course the terms used in consciousness studies—sentience, awareness, subjectivity, affects, feelings—cannot be precise, and are, no doubt, used differently by different scholars. For me the simplest and easiest is the word “experience”—namely certain brain states feel like something subjectively, and thus deserve to be called phenomenally conscious. Of course, others may only choose to use the term conscious, when animals can be shown to be “aware of” (can think and reflect upon) their experiences. I think that is too biased and shortsighted a view.

If one uses the concept of *consciousness* phenomenally, anchored simply by the existence of *subjective experiences*, it seems likely that primary-process consciousness comes in two major varieties—cognitive (linked to exteroceptive, perception-generating sensory inputs) and affective (internal states that feel good and bad in distinct ways). If so, as we consider the evolutionary layering of the BrainMind (Figure 2), we should recognize that affective functions are more medial in the brain than external perceptual ones, suggesting that affect is more ancient, and hence would have had priority in the construction of the mental apparatus. Perhaps the term “awareness” should be reserved just for higher forms of perceptual consciousness. By my wits, sensory perceptions, in some currently unknown way, may have arisen from the pre-existing neural platform for affective neurodynamics [2], [6], [43]. If so, affective experiential states may still be independent of the *cognitive knowledge* that you are experiencing such brain states.

In closing, I would note that just as the final revision of this manuscript was completed, a fine paper on this topic by Franz de Waal appeared [67] which presents a compelling argument for scientists to develop a renewed interest in emotions but in ways that “avoid unanswerable questions and to view emotions as mental and bodily states that potentiate behavior appropriate to environmental challenges” (p. 191). In this paper de Waal provides a compelling argument for the importance of animal emotions, while not crossing the Rubicon to discussions of emotional experiences.

As de Waal now expresses, in a toned down way compared to an earlier version of the manuscript (I was a reviewer), we can study animal emotions “without knowing much of anything about associated experiences” (p. 199) and that “the greatest obstacle to the study of animal emotions is the common objection that “we cannot know what they feel.” While this is undeniably true, we should realize that such problems also hold for fellow human beings (p. 199). But affective neuroscience strategies now provide the needed “weight of evidence” indicating that animals do “feel” although, admittedly, we cannot be very precise about the experienced nature of their feelings, above and beyond several distinct forms of good and bad emotional feelings. But how their brains allow them to feel good and bad in various ways will, one day, inform us scientifically, for the first time, about the nature of our own feelings. The cross-species ethical consequences of this knowledge, although intuited by many, are huge.

## Supporting Information

Appendix S1 (DOC)
