# Supplementary material for: Cross-Species Affective Neuroscience Decoding of the Primal Affective Experiences of Humans and Related Animals
Source: PLoS One. 2011 Sep 7;6(9):e21236. doi: 10.1371/journal.pone.0021236 (PMC3168430; doi:10.1371/journal.pone.0021236)
Supplement: Appendix S1 — (DOC) [file pone.0021236.s001.doc]

**APPENDIX S1: Summaries of Primary Process Emotional Systems**

The following are the primary-process emotional operating systems that have been identified by localized electrical stimulation of the brain. This list does not imply that there are no other affects. For instance, there are diverse sensory pleasures and displeasures, but they are not appropriately placed in the *emotion* category, for they are not dependent on “moving out” dynamically to engage the environment in emotion characteristic ways.  There are also various *homeostatic* affects, for instance hunger and thirst, that reflect the ability of the brain to monitor body state variables that are important for survival, but all of these operate through one major emotional system, the SEEKING system, to be described first. Detailed summaries of these emotional systems are available elsewhere , and a few additional recent specific summaries of each system are provided.

These sketches start with the most pervasive emotional system of them all (SEEKING), followed by three reptilian emotions (RAGE, FEAR and LUST), and then the social emotions well developed in mammals (CARE, PANIC/GRIEF, and PLAY). Again, the capitalizations reflect a nomenclature convention for designating primary-process emotional systems. Neuroanatomical and neurochemical details of these systems are summarized in Figure 5.

1) The SEEKING system facilitates all appetitive desires. It provokes animals to find and harvest the fruits of the world.  Animals readily self-activate this brain system—they ‘self-stimulate’ this circuitry in compulsive, addictive ways.  Indeed, this system is important for all varieties of drug addictions and also for addictive behaviors ranging from gambling to hoarding, with many others in-between, for instance, probably compulsive inter-netting and sexuality. This system is still, after half a century, called “The Brain Reward System” even though there are many rewarding systems in the brain. However, there is emerging agreement that this system does not mediate “liking” of rewards but rather the “wanting” of them. . . including resources such as “safety”. Thus, this system also helps the RAGE and FEAR systems achieve their appointed tasks. However, SEEKING is largely an action-organizing system, generating approach-exploratory behaviors at the very lowest level and desires and aspirations at the highest levels (for a recent synopsis of views concerning this system, see ).  With learning, diverse forms of cognitive information descend into this system, but the output is much less resolved, coaxing the animal to behave in appetitively aroused, goal-directed ways. In any event, this system highlights the massive degree to which a basic state control system that mediates the primary-process phenomenology of appetitive urges can readily link up with cognitive systems that mediate secondary-process learning and tertiary-process forms of awareness and appraisals (for a comprehensive summary, see ).

2) The RAGE system provokes animals to protect their resources. It is activated by enforced bodily restraint (i.e., restriction of freedom of action) as well as the perception that other life-supporting resources are may be taken away. Arousal of this neural system is aversive, and it highlights the hierarchical organization of most primary-process emotional systems, where lower-brain functions, such as the periaqueductal gray (PAG) of the midbrain, are essential for intermediate brain controls, emanating from the hypothalamus, and higher controls such as corticomedial amygdala. Those higher control aspects of the primary-process circuitry cannot work effectively without the lower components of the circuitry.

3) The FEAR system specifically organizes natural danger-escape and avoidance behavior patterns such as freezing and flight that allow animals to stay out of harm’s way. There are only a few unconditioned stimuli that can access this system prior to learning—for instance the smell of predators (e.g., cats for rats and mice, and loss of physical support and unprotected heights in humans). Neuroscientists have unraveled some of the intrinsic details of FEAR circuitry (Figure 5) which runs from the central nucleus of amygdala to the PAG of the midbrain. Even more work has been done on how this system comes under the control of learned inputs, and there is abundant speculation on how it participates in various psychiatrically significant anxiety disorders. For a recent summary, see .

4) Reproducing depends on well-functioning LUST systems . Male and female sexual systems are laid down early in development, while babies are still gestating, but they are not brought fully into action until puberty, when the maturing gonadal hormone systems begin to spawn male and female sexual desires.  However, because of the way the brain and body get organized, female-type desires can exist in male brains, and male-type desires can exist in female brains. Of course, learning and culture persistently add layers of control and complexity that cannot yet be disentangled by neuroscience. For overview, see .

5) Without a maternal CARE system , the young of all mammalian species would die at an early age, insuring species extinction. The maternal instinct, so rich in every species of mammal (and bird too), allows inter-generational support needed for highly social species to propagate effectively down generations. This critical survival function is built into ancient regions of the brain, and provides the motivation for mother-infant social attachments. The system is spontaneously activated by changing tides of peripheral estrogen, progesterone, prolactin and brain oxytocin, which transform virgin female brains into fully maternal ones. This system is typically more robust in adult females and males. For a full description, see .

6) Young, helpless children exhibit a PANIC/GRIEF response when they get lost. This negative feeling helps cement infant-mother attachments. Young animals are adept at crying out for care, and such feeling of sudden social loss is a separation-distress process—a psychic pain—that appears to be based on a brain system that eventually can precipitate adult sadness and grief.  The anatomies of this system appear to be of great importance for psychiatric disorders such as depression. Precipitous arousal of this system may be essential for panic attacks. This system may also contribute to childhood disorders such as autism. For recent review, see .

7) The social PLAY of young animals reflects the operation of a primary process emotional system that allows young animals to explore diverse social possibilities in joyous ways and to develop friendships and form social alliances.  The urge to play was not left to chance by evolution, but is built into the instinctual action apparatus of the mammalian brain, and PLAY motivation in animals is signaled by sounds that resemble laughter in our species . Just like the other systems described above, PLAY survives radical decortication—complete removal of the neocortical mantle that covers the upper brain stem . If young children are not allowed enough play, they may exhibit more impulsive, acting-out behaviors that often get them labeled as troublemakers (e.g., with Attention Deficit Hyperactivity Disorder); all psychostimulant drugs used to treat ADHD reduce playfulness in animals .

1. Panksepp J (1998) Affective neuroscience: The foundations of human and animal emotions. New York: Oxford University Press.

2. Panksepp J (2005) Affective consciousness: Core emotional feelings in animals and humans. Consciousness and Cognition 14: 30-80.

3. Panksepp J, Biven L (2011) The archaeology of mind: Neuroevolutionary origins of human emotion: Norton, W. W. & Company, Inc.

4. Panksepp J, Moskal J (2008) Dopamine and SEEKING: Sub-neocortical “reward” systems and appetitive urges. In: Elliot A, editor. Handbook of approach and avoidance motivation. New York: Taylor & Francis Group, LLC. pp. 67-87.

5. Alcaro A, Huber R, Panksepp J (2007) Behavioral functions of the mesolimbic dopaminergic system: An affective neuroethological perspective. Brain Research Reviews 56: 283-321.

6. Alcaro A, Panksepp J, Witczak J, Hayes DJ, Northoff G (2010) Is subcortical-cortical midline activity in depression mediated by glutamate and GABA? A cross-species translational approach. Neuroscience and Biobehavioral Reviews 34: 592-605.

7. Siegel A (2005) The neurobiology of aggression and rage. Boca Raton, FL: CRC Press.

8. Guerra DJ, Colonnello V, Panksepp J (2010) The neurobiology of rage and anger & psychiatric implications with a focus on depression. In: Pahlavan F, editor. Multiple facets of anger: Getting mad or restoring justice? New York: Nova Science Publishers, Inc. pp. 81-103.

9. Panksepp J, Fuchs T, Iacobucci P (2011) The basic neuroscience of emotional experiences: The case of FEAR and implications for clinical anxiety in animals and humans. Applied Animal Ethology 129: 1-17.

10. Pfaff DW (1999) Drive: Neurobiological and molecular mechanisms of sexual behavior. Cambridge MA: MIT Press.

11. Neuman M, Insel TR (2003) The neurobiology of maternal behavior. New York: Springer.

12. Panksepp J (2011) The neurobiology of social loss in animals: Some keys to the puzzle of psychic pain in humans. In: MacDonald G, Jensen-Campbell LA, editors. Social pain: Neuropsychological and health implications of loss and exclusion. Washington D.C.: American Psychological Association. pp. 11-51.

13. Watt DF, Panksepp J (2009) Depression: an evolutionarily conserved mechanism to terminate separation-distress? A review of aminergic, peptidergic, and neural network perspectives. Neuropsychoanalysis 11: 5-104.

14. Pellis S, Pellis V (2009) The playful brain: Venturing to the limits of neuroscience. Oxford, UK: Oneworld Pubs.

15. Panksepp J (2010) The science of the brain as a gateway to understanding play. American Journal for Play 4: 245-277. (This whole issue of AJP is devoted to summaries of the neuroscience of play)

16. Burgdorf J, Panksepp J (2006) The neurobiology of positive emotions. Neuroscience and Biobehavioral Reviews 30: 173-187.

17. Burgdorf J, Wood PL, Kroes RA, Moskal JR, Panksepp J (2007) Neurobiology of 50-kHz ultrasonic vocalizations in rats: Electrode mapping, lesion, and pharmacology studies. Behavioural Brain Research 182: 274-283.

18. Panksepp J, Normansell L, Cox JF, Siviy SM (1994) Effects of neonatal decortication on the social play of juvenile rats. Physiology & Behavior 56: 429-443.

19. Panksepp J (2007) Can PLAY diminish ADHD and facilitate the construction of the social brain? Journal of the Canadian Academy Child Adolescent Psychiatry 16: 57-66.
